# Assessment of trends in socioeconomic inequalities in cancer screening services in Korea, 1998–2012

**DOI:** 10.1186/s12939-016-0319-7

**Published:** 2016-02-24

**Authors:** Sujin Kim, Jongnam Hwang

**Affiliations:** Takemi program in International Health, Harvard School of Public Health, Boston, MA USA; Centre for Research on Inner City Health, St. Michael’s Hospital, 30 Bond Street, Toronto, ON M5B 1 W8 Canada

**Keywords:** Cancer screening, Inequality, Concentration index, Decomposition, Korea, Preventive care

## Abstract

**Background:**

This study aimed to examine how income-related inequalities in screening services for gastric and colorectal cancer in Korea have changed over the past decades, along with the implementation of the national cancer screening program, and also to quantify each contribution from various socio-demographic factors income-related inequalities with respect to these cancer screening services.

**Methods:**

Three cycles (1998, 2005, and 2010–2012) of Korea National Health and Nutrition Examination Survey (KNHANES) were utilized. To measure income-related inequalities in the use of gastric and colorectal cancer, individuals over the age of 40 and the age of 50 were included respectively, and the Concentration Index (CI) was calculated for each cycle. To identify and quantify contribution from each socio-demographic factor, decomposition of the CIs was conducted.

**Results:**

Throughout this study, CIs and horizontal inequity indices (HIs) steadily but consistently decreased, suggesting that inequalities and inequities in participation in gastric and colorectal cancer screening were weakened after the implementation of the national public cancer screening program. Decomposition analyses revealed that whereas decreases in inequalities mostly stemmed from income and educational levels; higher income and better education levels are still major contributors to the observed inequalities that influence participation in cancer screening services in Korea.

**Conclusion:**

Our empirical findings suggest that, although the policy of reducing out-of-pocket payment for cancer screening may contribute to the observed decreases in inequality, it alone is not likely to completely eliminate inequality. Further research is required to identify barriers that prevent people with lower socioeconomic status from participation in cancer screening, which allows equal access for equal need.

**Electronic supplementary material:**

The online version of this article (doi:10.1186/s12939-016-0319-7) contains supplementary material, which is available to authorized users.

## Background

Cancer screening refers to the use of simple tests across a healthy population to identify individuals who have the disease [1]. It contributes to considerable reduction of both clinical and financial burdens of through early detection and timely treatment for such as colorectal, cervical, and breast cancers [2–4]. For example, the decreased incidence of colorectal cancer during the 1980s and 1990s in the United States is known to be attributed to an increase in screening services [5]. In Korea, patients screened for gastric cancer appeared to have 30–70 % lower mortality compared to un-screened patients [6]. Despite the significant clinical benefits of preventive cancer screening services, a concern arises that the rate of screening services are not uniformly utilized over the population [7, 8]. Cumulative evidence suggests that participation in screening is more concentrated in individuals with higher income for various cancers such as breast, cervical, colorectal and gastric [8–11].

To increase screening and reduce the imbalance in a population, a population screening program at the national level, which establishes guidelines for age-specified regular cancer screenings, is recommended [2]. Nation-wide screening programs are closely related to overall increases in the use of cancer screening services and decreases in cancer incidence and cancer-related mortality [2]. It is not clear, however, whether these programs facilitate equal use of such services across different income groups [7]. Few studies have examined the impact of the implementation of public cancer screening programs, observing that national-level mass screening may contribute to decreasing inequality in screening participation for breast and cervical cancers [12–16]. Existing evidence is somewhat limited; therefore, the impact of those mass screening policies across socioeconomic status (SES) is not fully understood.

In Korea, the national cancer screening program was implemented in 1999 to provide free screening services for gastric, cervical, and breast cancer to Medicaid enrollees (means-tested program for individuals of lower income); eligible individuals at a certain age receive a letter including detailed information about overall cancer screening procedures. Later, the program expanded the target population to include more low-income people along with Medicaid enrollees; additionally, it included colorectal cancer as a target cancer in 2004 [17]. As of 2006, the program provides free screening services for those in the bottom 50 % of income groups and subsidizes 80 % of the costs for high income groups [17]. Since the implementation of mass screening programs, the rate of cancer screening has steadily increased in Korea [13, 18, 19]; however, differences across income groups receiving the screening are still observed, with 41 % in the lowest income quartile receiving screening compared with 54 % in the highest in 2012 [20].

As there is limited evidence on whether or not the program contributes to encouraging individuals with lower income to utilize the screening, it still remains unanswered if the current mass screening program supports diminishing income-related inequality in cancer screening services. Therefore, this study aimed to examine whether income-related inequalities in cancer screening services exist, and if so, whether the trends of the inequalities has shrunk since the implementation of the national cancer screening program in Korea, 1998–2011. Furthermore, this study assessed the contribution of various socio-demographic factors to the observed inequalities in the use of cancer screening services over the past years.

## Methods

### Data source

Data from Korea National Health and Nutrition Examination Survey (KNHANES) from 1998 (Cycle 1), 2005 (Cycle 3), 2010–2012 (Cycle 5) were utilized. The KNHANES is a nationally representative cross-sectional survey for examining health and nutritional status of Koreans and monitoring health risk factors and the prevalence of chronic disease in the Korean population [21]. Starting from the 2007 survey (Cycle 4), the frequency of the survey has been changed from once every 3 year to every year, and cycle 4 and 5 are composed of a three-year survey [21]. The KNHANES is comprised of non-institutionalized Koreans residing in Korea who are sampled based on a multi-stage clustered probability design, and the survey collects detailed information on socio-demographic information, health behaviours, quality of life, healthcare utilization, and results of various health examination [21]. The KNHANES survey weights are provided in order to represent the entire Korean population by adjusting for complex survey designs, survey non-response and post-stratification [21]. The KNHANES is statistically designed for comparisons between different cycles, and any study using the KNHANES data is generally recommended applying KNHANES survey weights [22]. More information on the KNHANES can be found at http://knhanes.cdc.go.kr/knhanes/eng.

In this study, we included respondents over the age of 40 for gastric cancer (*n* = 4819 for cycle 1 (1998), 4580 for cycle 3 (2005), 12274 for cycle 5 (2010–2012)) and over the age of 50 for colorectal cancer (*n* = 2887 for cycle 1(1998), 2610 for cycle 3 (2005), 8460 for cycle 5(2010–2012)), based on the screening initiation age for each cancer recommended by the national cancer screening program in Korea [17].

### Variables

#### Gastric and colorectal cancer screening

The use of cancer screening services was measured by an individual’s self-report as to whether they had received gastric and colorectal cancer screening services. In the KNHANES, each participant was asked *“Have you received gastric/colorectal cancer screening services within the past two years?”* and the participant recorded their response as *“Yes or No”*.

#### Socioeconomic status

To measure the degree of inequality in gastric and colorectal cancer screening participation, equivalised annual household income was used. Income has been considered as a main indicator of SES, and it has been widely used for Concentration Index (CI) and decomposition analysis [23, 24].

#### Other variables

For our decomposition models, socio-demographic factors determining participation in cancer screening were selected in order to quantify each contribution of the factors to the observed inequality. These socio-demographic factors were selected based on Andersen’s Health Behaviour Model and determinants of health care utilization from previous studies [25–27]. The Andersen’s Health Behaviour Model is a conceptual model introducing a wide range of factors associated with use of health care services. In this model, the use of health services is determined by three dimensions including predisposing factors (e.g., age, gender, education, employment status, etc.), enabling factors (e.g., income, health insurance, and a regular source of health care, etc.), and need factors (e.g., objective and subjective needs). Based on included various socio-demographic variables for decomposition analysis, we identified need and non-need factors. The need factors (i.e., age (continuous), sex, and self-rated health (good; fair; poor)) generally reflect an individual’s health care needs, representing difference in need of health services. The non-need factors include various socioeconomic factors such as marital status (single; married), educational level (elementary school; middle school; high school; university & above), employment status (manual; non-manual; others including unemployment and out of labour market), income (quintile), region (metro Seoul areas; non-metro Seoul areas), place of residence (urban; rural) and type of national health insurance (NHI, Medicaid; neither).

### Statistical analyses

To measure socioeconomic inequalities in participation in cancer screening services for 1998 (Cycle 1), 2005 (Cycle 3), and 2010–2012 (Cycle 5), we first calculated the CI for each cycle of the KNHANES, a tool which has been widely used to assess income-related inequality in the fields of health economics and policy research [28, 29]. After we obtained the CIs, we employed decomposition approaches to quantify socio-demographic factors contributing to the observed inequalities and its changes in contribution of socio-demographic factors over the past 13 years in relation to both gastric and colorectal screening services in Korea.

#### Concentration index

The CI is defined as twice the area between the line of equality (the 45-degree line) and a concentration curve, where the individuals are ranked by socioeconomic levels, generally income and the cumulative rankings of each individual is plotted against the cumulative share of health outcomes or healthcare utilization [30]. The CI represents whether the outcome variable of interest is concentrated more at lower of higher level of income. The outcome variable is concentrated among the rich (the poor), the CI has a positive (negative) value, suggesting “pro-rich” (“pro-poor”). The CI can be expressed as:

$$ \complement =\frac{2}{\mu }cov\left({y}_i,{r}_i\right) $$

Where *y* is the health care variable, *r* is the fractional rank in the income distribution and $\mu$ is the mean of the health care variable. The CI is typically bound between -1 and +1; however, for binary outcome, the bounds of the CI depend on the minimum, and the maximum, and the mean of the outcome variable. When the CI is used to compare inequality across time, place and sub-population group, calculating the CI for binary outcome is potentially problematic because the possible range of the CI value differs by mean of the outcome variable [31–34]. To resolve this issue, the CI needs to be normalized by multiplying (1- mean of the outcome variable), as it referred normalized CI in this study, following the prior studies [31–33].

#### Decomposition of the CI and calculating Horizontal inequity index (HI)

The basic idea of decomposing the CI is quantifying each contribution of need and non-need factors to the observed CI because a sum of the contribution from each factor and residuals is the overall CI [35]. Decomposition of the CI can be expressed by the formula:

$$ C={\displaystyle {\sum}_k}\left(\frac{\beta_k{\overline{x}}_k}{\mu}\right){c}_k+\frac{C{G}_{\varepsilon }}{\mu } $$

Where the index *K* refers to the regressor included in the underlying equation, *C*_κ_ is the CI for each of the individual regressor, β_κ_ is the coefficient for each of the determinants, ${\overline{x}}_k$ is the mean of each of the regressor, and *μ* is the mean of the health care variable under consideration. *CG*_ε_ is the generalized C for the residual from the underlying regression [35]. The residual error term in this equitation represents the inequality in the use of cancer screening services that is not explained by systematic differences [35].

Followed by decomposing the CIs, Horizontal inequity index (HI) was obtained by subtracting all contributions from “need” factors from the overall CI [35]. The HI is a widely used tool for determining whether or not the observed inequality can be considered as a matter of inequity [35]. A positive (negative) HI indicates a higher share of health care use among the better-off (the worse-off). The HI is based on the idea that access to health services is equitable when individuals have the equal need of the service [35]. Inequality and inequity are used interchangeably in existing literature, but a distinction should be made [36]. While both inequality and inequity indicate difference in health or health care between different population groups, the latter one implies unfairness or injustice that should be modified [36, 37].

#### Decomposition of changes in the CI

To understand how each variable contributes to changes in the CI between cycle 1 (KNHANES 1998) and cycle 5 (KNHANES 2010–2012), an Oaxaca-type decomposition of the CI was applied.

$$ C 2\ \hbox{--}\ C 1={\displaystyle {\sum}_k{\eta}_{kt2}\left({C}_{kt2}-{C}_{kt1}\right)+{\displaystyle {\sum}_k{C}_{kt1}\left({\eta}_{kt2}-{\eta}_{kt1}\right) + \left(\frac{G{C}_{\varepsilon t2}}{\mu_{t2}}\right)-\left(\frac{G{C}_{\varepsilon t1}}{\mu_{t1}}\right)}} $$

Where *C1* and *C2* are the CI for two different cycles- cycle1 (KNHANES 1998) and cycle 5 (KNHANES 2010–2012), ${\eta}_{kt}$ is the elasticity for the *k* regressor at time t (t1:1998 and t2: 2010–2012), ${C}_{kt}$ is the CI of determinants *k* at time t and the last two terms constitute the difference in the residuals from decomposition of cycle1 (KNHANES 1998) and cycle 5 (KNHANES 2010–2012) [23].

All analyses were conducted using STATA v. 12, and survey weights provided by KNHANES were applied to all analyses.

## Results

Table 1 shows the total numbers of cancer screening recipients in 1998 (Cycle 1), 2005 (Cycle 3) and 2010–2012 (Cycle 5). The average rate of colorectal and gastric cancer screening over 14 years was 30.4 % and 40.5 %, respectively. The screening rates in both cancer screenings continuously increased, from 7.4 % in 1998 to 42.0 % in 2010–2012 for colorectal cancer and from 13.7 % in 1998 to 54.0 % in 2010–2012 for gastric cancer. The descriptive characteristics of the respondents by cancer screening services are found in Additional file 1 (Tables S1 and S2).

**Table 1:** A total sample of cancer screening recipients by KNHANES cycle 1 (1998), cycle 3 (2005), cycle 5 (2010–2012)^*^

Colorectal cancer	Yes	%	No	%	Total
Cycle 1 (1998)	214	7.4	2663	92.6	2877
Cycle 3 (2005)	475	18.2	2135	81.8	2610
Cycle 5 (2010–2012)	3556	42.0	4904	58.0	8460
Gastric cancer	Yes	%	No	%	Total
Cycle 1 (1998)	660	13.7	4159	86.3	4819
Cycle 3 (2005)	1490	32.5	3090	67.5	4580
Cycle 5 (2010–2012)	6624	54.0	5650	46.0	12274

^*^The KNAHNES response rate: cycle 1 (1998): 85.6 %; cycle 3 (2005): 92.8 %; cycle 5 (2010–2012): 80.8 %

**Fig. 1:**
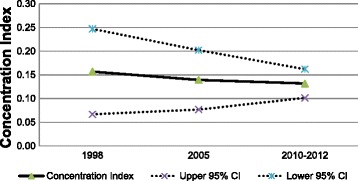
Concentration Index^*^ for colorectal cancer screening services with 95 % confidence interval, Korea National Health and Nutrition Examination Survey (KNHANES) cycle 1 (1998), cycle 3 (2005), and cycle 5 (2010–2012). *Concentration index presented in this figure are normalized concentration indices (Wagstff, 2005;2011)

**Fig. 2:**
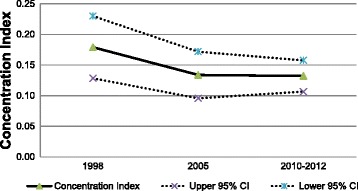
Concentration Index^*^ for gastric cancer screening services with 95 % confidence interval, Korea National Health and Nutrition Examination Survey (KNHANES) cycle 1 (1998), cycle 3 (2005), and cycle 5 (2010–2012). *Concentration index presented in this figure are normalized concentration indices (Wagstff, 2005;2011)

The concentration indices (CIs) for cancer screening services are shown in Fig. 1 and Fig. 2. Positive values of the CI observed consistently over time indicates that more use of cancer screening services has been concentrated among individuals with higher income for both cancers, but the degree of the CIs has slightly decreased for gastric cancer from 0.179 (95 % CI: 0.128–0.230) in 1998 to 0.132 (95 % CI: 0.107–0.158) in 2010–2012. The CIs for the use of colorectal cancer screening also have decreased to 0.131 (95 % CI: 0.102–0.162) in 2010–2012 from 0.157 (95 % CI: 0.067–0.247) in 1998.

Table 2 and Table 3 present the decomposition of the CIs in colorectal cancer screening services and gastric cancer screening services for 1998 and 2010–2012, respectively. Elasticity for each factor ($\frac{\beta_k{\overline{x}}_k}{\mu}\Big)$ in the first column shows the sensitivity of cancer screening services with respect to each factor. The CI for each factor is presented in the second column. This indicates the distribution of each factor by income levels. For instance, positive C_*k*_ value (second column of each year) for highest education (Education – completion of University or above) in Tables 2 and 3 indicates that highest educational attainment is more concentrated in the rich. Lastly, contribution ($\left(\frac{\beta_k{\overline{x}}_k}{\mu}\right)$ C_*k*_) in the third column shows how much each factor contributes to inequality (the overall CI) in the use of cancer screening services. Positive contribution of a certain factor shows the factor contributes to the measured “pro-rich” inequalities in cancer screening services. For colorectal cancer screening, highest income quintile (income quintile 5) was consistently the largest contributor to the overall CIs over the past years (Table 2). Higher education attainment (completion of high school, and university or above) was the second largest contributor to the pro-rich patent of the CI in 1998 and in 2010–2102 (Table 2).

**Table 2:** Results from decomposition analysis for participation of colorectal cancer screening services among Korean adults, Korea National Health and Nutrition Examination Survey (KNHANES) cycle 1 (1998), cycle 3 (2005), and cycle 5 (2010–2012)

Colorectal cancer screening	Cycle 1 (1998)			Cycle 3 (2005)			Cycle 5 (2010–2012)		
	Elasticity	C_k_	Contribution	Elasticity	C_k_	Contribution	Elasticity	C_k_	Contribution
Sex- female	0.039	-0.016	-0.001	-0.198	-0.024	0.005	-0.009	-0.024	0.000
Age	-0.636	-0.017	0.011	-0.083	-0.029	0.002	-0.264	-0.039	0.010
Self-rated health - fair	-0.076	0.102	-0.008	-0.088	0.088	-0.008	0.037	0.040	0.001
Self-rated - poor	-0.097	0.033	-0.003	-0.094	0.176	-0.017	0.009	0.127	0.001
Sub-total - need factors			-0.001			-0.017			0.013
Marital status- married	0.030	0.041	0.001	-0.056	0.082	-0.005	0.113	0.090	0.010
Education - completion of middle school	0.024	0.142	0.003	0.060	0.120	0.007	0.026	0.090	0.002
Education - completion of high school	0.138	0.263	0.036	0.012	0.232	0.003	0.033	0.222	0.007
Education - completion of university or above	0.045	0.501	0.023	0.010	0.397	0.004	0.019	0.400	0.008
Employment - non-manual job	0.014	0.760	0.011	-0.003	0.657	-0.002	0.003	0.493	0.001
Employment-others^a^	0.039	-0.177	-0.007	-0.054	-0.240	0.013	0.010	-0.160	-0.002
Income quintile 2 (low)	0.118	-0.349	-0.041	0.062	-0.319	-0.020	0.027	-0.447	-0.012
Income quintile 3	0.032	0.031	0.001	0.045	0.060	0.003	0.039	-0.057	-0.002
Income quintile 4	0.037	0.508	0.019	0.103	0.401	0.041	0.028	0.366	0.010
Income quintile 5(high)	0.099	0.910	0.090	0.112	0.830	0.093	0.039	0.799	0.031
Health insurance - National Health Insurance	-0.294	0.030	-0.009	0.239	0.042	0.010	0.106	0.025	0.003
Region - Metro Seoul areas	0.061	0.159	0.010	0.020	0.156	0.003	0.013	0.076	0.001
Place of residence - rural areas	-0.247	-0.052	0.013	0.143	-0.044	-0.006	-0.151	-0.033	0.005
Sub-total - non-need factors			0.150			0.144			0.063
Residual			0.008			0.012			0.055
Normalized concentration index (CI) (95 % confidence interval)		0.157 (0.067–0.247)			0.139 (0.077–0.202)			0.131 (0.102–0.162)	

^a^“Others” included unemployment and out of labour market

**Table 3:** Results from decomposition analysis for participation of gastric cancer screening services among Korean adults, Korea National Health and Nutrition Examination Survey (KNHANES) cycle 1 (1998), cycle 3 (2005), and cycle 5 (2010–2012)

Gastric cancer screening	1998			2005			2010–2012		
	Elasticity	C_k_	Contribution	Elasticity	C_k_	Contribution	Elasticity	C_k_	Contribution
Sex- female	-0.072	-0.015	0.001	0.031	-0.020	-0.001	0.097	-0.018	-0.002
Age	-0.250	-0.037	0.009	-0.169	-0.050	0.008	0.295	-0.052	-0.015
Self-rated health - fair	-0.079	0.090	-0.007	-0.045	0.069	-0.003	0.026	0.026	0.001
Self-rated - poor	-0.132	0.041	-0.005	-0.069	0.172	-0.012	0.015	0.119	0.002
Sub-total - need factors			-0.002			-0.007			-0.014
Marital status- married	0.086	0.060	0.005	0.101	0.097	0.010	0.145	0.084	0.012
Education - completion of middle school	0.026	0.034	0.001	0.013	-0.013	0.000	0.017	-0.049	-0.001
Education - completion of high school	0.084	0.185	0.016	-0.032	0.144	-0.005	0.025	0.133	0.003
Education - completion of university or above	0.077	0.463	0.036	-0.002	0.431	-0.001	0.018	0.347	0.006
Employment - non-manual job	0.051	0.564	0.029	0.031	0.568	0.017	0.013	0.382	0.005
Employment - others^a^	0.073	-0.120	-0.009	0.003	-0.313	-0.001	-0.012	-0.185	0.002
Income quintile 2 (low)	0.079	-0.424	-0.033	0.063	-0.366	-0.023	0.019	-0.468	-0.009
Income quintile 3	0.053	-0.063	-0.003	0.033	0.040	0.001	0.022	-0.053	-0.001
Income quintile 4	0.142	0.420	0.060	0.037	0.404	0.015	0.039	0.384	0.015
Income quintile 5(high)	0.066	0.869	0.058	0.106	0.821	0.087	0.044	0.810	0.036
Health insurance - National Health Insurance	-0.034	0.025	-0.001	-0.181	0.036	-0.007	0.031	0.024	0.001
Health insurance - neither	-0.008	-0.327	0.003	-0.002	-0.565	0.001	0.000	-0.184	0.000
Region - Metro Seoul areas	0.031	0.122	0.004	0.016	0.113	0.002	-0.009	0.044	0.000
Place of residence - rural areas	-0.116	-0.063	0.007	0.095	-0.038	-0.004	-0.005	-0.033	0.000
Sub-total - non-need factors			0.170			0.094			0.060
Residual			0.011			0.047			0.077
Normalized concentration index (CI) (95 % confidence interval)		0.179 (0.128-0.230)			0.134 (0.096-0.172)			0.132 (0.107-0.158)	

^a^“Others” included unemployment and out of labour market

Higher income (income quintile 4 and 5) was the major contributor to the existing inequality in the use of gastric cancer screening services (Table 3). Completion of university or above and non-manual job also positively contributed to the pro-rich overall CIs in 1998 and 2010–2012 (Table 3).

**Fig. 3:**
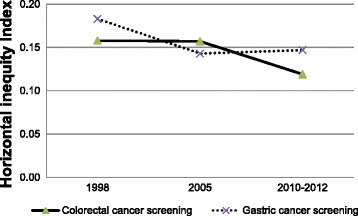
Horizontal inequity index (HI) for colorectal and gastric cancer screening services, Korea National Health and Nutrition Examination Survey (KNHANES) cycle 1 (1998), cycle 3 (2005), and cycle 5 (2010–2012)

For horizontal inequity index (HI), obtained by subtracting a sub total contribution of need factors from the overall CI, positive values of HIs for colorectal cancer screening services were consistently observed in the past years (1998: 0.158; 2005:0.157; 2010–2012: 0.118). Although the degree of the HI has steadily decreased, inequity in the use of colorectal cancer screening still persisted (Fig. 3). For the use of gastric cancer screening, a similar pattern was observed, suggesting a persistence of inequity between 1998 (HI: 0.182) and 2010–2012 (HI: 0.146), but the magnitude has become relatively smaller compared to the year of 1998 (Fig. 3).

To understand what factors have contributed to changes in the CIs in the use of both cancer screening services between cycle 1 (1998) and cycle 5 (2010–2012), we conducted Oaxaca type-decomposition for changes in the CI as an additional analysis. Tables 4 and 5 show the changes in the CIs in colorectal and gastric cancer screening services. The first column shows changes in magnitude of inequality in the contributors and the second column shows changes in the elasticity of the cancer screening services with respect to these contributors. The total changes for each determinant and the percent changes are presented in the last two columns. Overall, changing elasticity than changing CIs were more likely to contribute to the reduction in the pro-rich inequalities in cancer screening services. For colorectal cancer screening services, differences in higher education, and higher income were largely responsible for a decrease in inequality. With respect to gastric cancer screening services, the largest determinants for changes in inequality were also from income and education levels. It is interesting to note that being out of labor market and unemployed contributed to increasing inequalities in gastric and colorectal cancer screening services.

**Table 4:** Oaxaca-type decomposition for changes in the CIs in colorectal cancer screening services between Korea National Health and Nutrition Examination Survey (KNHANES) cycle 1(1998) and cycle 5 (2010-2012)

Oaxaca-type approach for colorectal cancer screening	Between cycle 1(1998) and cycle 5 (2010-2012)			
	Changes arising from change in			
	C_k_	Elasticity	Total	Percentage
Sex- female	0.0001	0.0008	0.0009	-3.33
Age	0.0058	-0.0062	-0.0004	1.50
Self-rated health - fair	-0.0024	0.0116	0.0092	-35.88
Self-rated - poor	0.0009	0.0035	0.0044	-17.14
Marital status- married	0.0056	0.0034	0.0090	-35.20
Education - completion of middle school	-0.0014	0.0003	-0.0010	3.98
Education - completion of high school	-0.0014	-0.0275	-0.0288	112.44
Education - completion of University or above	-0.0019	-0.0134	-0.0153	59.48
Employment - non-manual job	-0.0007	-0.0085	-0.0093	36.19
Employment - others^a^	0.0002	0.0051	0.0052	-20.40
Income quintile 2 (low)	-0.0026	0.0317	0.0291	-113.51
Income quintile 3	-0.0034	0.0002	-0.0032	12.51
Income quintile 4	-0.0040	-0.0045	-0.0085	33.04
Income quintile 5(high)	-0.0043	-0.0545	-0.0588	229.18
Health insurance - National Health Insurance	-0.0005	0.0119	0.0114	-44.30
Region - Metro Seoul areas	-0.0010	-0.0077	-0.0088	34.27
Place of residence - rural areas	-0.0028	-0.0048	-0.0079	30.80
Sub-total	-0.0139	-0.0608	-0.0727	283.64
Residual			0.0471	-183.64
Total	-0.0251		-0.0256	100.00

^a^“Others” included unemployment and out of labour market

## Discussion

This study attempted to measure income-related inequalities in the use of gastric and colorectal cancer screening services by estimating the concentration index (CI), and also quantified each contribution from various socio-demographic factors such as educational and employment status to the observed inequalities by decomposing the CI over the past decades. The CIs for the use of cancer screening services appeared to decrease from 0.157 to 0.131 in colorectal cancer and from 0.179 to 0.132 in gastric cancer, which reveals a steady but consistent decline in income-related inequalities in participation in gastric and colorectal cancer screening services over times in Korea. In addition, Horizontal inequity Index (HI), indicating whether the services participation was equitable across the population after need-adjustment, also consistently persisted, suggesting that the observed inequalities between 1998 and 2010–2012 may be a matter of inequities, avoidable differences in participation in colorectal and gastric cancer screening services.

A plausible explanation for the observed decline in inequalities and inequities of both gastric and colorectal screening services is expanding coverage of free of charge screening services for larger population groups. Out-of-pocket (OOP) payments at the service offered in Korea’s National Health Insurance (NHI) have been widely reported as a major barrier to preventive health care services [38], and minimizing individual’s financial burdens by expanding the national cancer screening program may result in increasing accessibility of both cancer screening services, in particular among lower income groups.

Results from Oaxaca decomposition of the CIs reassure us that the contributor that is most responsible for decreasing inequalities is income. Income levels have been identified as a main factor driving unequal patterns of preventive cancer screening participation [26, 39]. Previous studies indicated that preventive screening programs, including nationwide population-based screening, is likely to facilitate utilization of the services from individuals with lower SES because the program minimizes possible barriers such as cost, time off work, and information [7, 40, 41]. The consistency of this finding implies that establishing or expanding public cancer screenings under universal health coverage may need to be considered when increasing participation rate of and reducing inequalities for individuals of lower SES.

Even though the CI and HI provide evidence on the degree of existing inequality and inequity with respect to the use of cancer screening services, a question related to its interpretation of the magnitude can been raised. To provide a better understanding of the values, we compared results with previous studies using the same methods. For instance, a recent UK study on colorectal cancer screening reported a pro-rich pattern with 0.16 of the CI value [27]. This value is similar to our 1998’s CI value but slightly larger than our 2010–2012’s CIs for both colorectal and gastric cancer screening services. Compared to other European countries such as Germany (0.126), France (0.135), and Belgium (0.04) [42], the CIs for both colorectal and gastric cancer screenings in Korea have become similar, but still show the persistence of inequalities and inequities. This may imply that the national screening program contributes to decreasing inequalities and inequities in participation in cancer screenings, whereas other national or institutional features may also account for these differences in the degree of inequality (inequity) in cancer screening. In fact, a previous study that measured the CIs in both public and private cancer screenings in Korea suggested different patterns of using cancer screening in different income groups; individuals with higher income were concentrated in the use of private cancer screening at their own cost (CI: 0.143) whereas individuals with lower income were more concentrated in use of public cancer screening services (CI:-0.118) [26, 39].

Although results from our additional analysis (Oaxaca- type decomposition for changes in the CIs) indicates that decreases in the inequalities stemmed from income and educational levels, results from the decomposition analyses in 2010–2012 reveal that higher income and better education levels still remain as major contributors to inequalities in participation in cancer screening services in Korea. Consistent with our study, a previous study investigating the utilization of cervical screening across different countries reported that income and education substantially contributes to causing inequalities despite the existence of national policy ensuring equal access or delivery of preventive services [2]. Also, it has been reported that less-educated women were less likely to utilize mammography even after the implementation of a Belgian national screening program that offers free mammography services, although there was an overall decrease of inequalities in cancer screening [14]. These findings from previous studies, along with our results, imply that the implementation of cancer screening programs at the national level could reduce the financial burden of individuals with lower income and educational levels, but it may fail to achieve equal utilization for all population groups.

To fully understand why inequalities still remain after the expansion of public cancer screening services in Korea, further investigation is required. One plausible explanation is that while an individual’s economic condition, mainly income, represents ability to pay for services, utilization of cancer screening services may be influenced by numerous factors correlated to income [3, 40, 43–46]. For example, people with lower income and precarious employment may have more difficulties in leaving work to seek preventive care services [47, 48]. Further, considering a profound relationship between income and educational level, lack of knowledge of health services -leading to low levels of health literacy- have an influence on informed decision making about screening [46, 49–51] as an individual may underestimate the risk of cancer and the benefits of preventive screening [7, 46, 49, 50, 52].

Because income appears to be such an important contributor, policy makers could further focus on understanding the income-cancer screening relationship if they aim to continually reduce the observed inequalities under the current public mass cancer screening program. According to our findings, a greater investment in public cancer screening at the national level is likely to increase not only the total rate of screening, but also can be potentially beneficial in diminishing income-related inequality in the uptake of cancer screening services. The recent national statistics indicates income inequality measured by Gini coefficient has steadily increased over the past decade [53]. This implies that income-related inequalities in participation of cancer screening services could become worse again if no effort to maintain or expand the current public cancer screening is made.

Nevertheless, it is worthwhile to caution that eliminating only financial barriers, without considering further policies to reduce inequality, could be more beneficial to higher SES [48]. In fact, our results from the Oaxaca-type decomposition analyses indicate that pro-rich patterns in those currently unemployed or out of labour market increased along with residual error indicating unexplained component by the included need and non-need factors for both screening services (Tables 4 and 5). Considering this result, policy actions for increasing cancer screening services among those currently unemployed and out of labour market need to be considered. Furthermore, other factors prohibiting individuals of lower income from undertaking screening services need to be further investigated.

**Table 5:** Oaxaca-type decomposition for changes in the CIs in gastric cancer screening services between Korea National Health and Nutrition Examination Survey (KNHANES) cycle 1(1998) and cycle 5 (2010-2012)

Oaxaca-type approach for gastric cancer screening	Between cycle 1(1998) and cycle 5 (2010-2012)			
	Change arising from change in			
	C_k_	Elasticity	Total	Percentage
Sex- female	-0.0003	-0.0025	-0.0028	5.81
Age	-0.0043	-0.0203	-0.0245	51.38
Self-rated health - fair	-0.0017	0.0095	0.0078	-16.35
Self-rated - poor	0.0012	0.0060	0.0072	-15.04
Marital status- married	0.0035	0.0035	0.0070	-14.57
Education - completion of middle school	-0.0014	-0.0003	-0.0017	3.53
Education - completion of high school	-0.0013	-0.0110	-0.0123	25.78
Education - completion of University or above	-0.0021	-0.0276	-0.0297	62.10
Employment - non-manual job	-0.0023	-0.0218	-0.0241	50.51
Employment - others^a^	0.0008	0.0102	0.0109	-22.90
Income quintile 2 (low)	-0.0008	0.0255	0.0247	-51.65
Income quintile 3	0.0002	0.0019	0,0021	-4.48
Income quintile 4	-0.0014	-0.0433	-0.0448	93.73
Income quintile 5(high)	-0.0026	-0.0190	-0.0216	45.23
Insurance - National Health Insurance	0.0000	0.0016	0.0016	-3.34
Insurance - neither	0.0000	-0.0026	-0.0026	5.54
Region - Metro Seoul areas	0.0007	-0.0048	-0.0041	8.64
Place of residence - rural areas	-0.0001	-0.0066	-0.0068	14.45
Sub-total	-0.0120	-0.1017	-0.1109	232.25
Residual			0.0632	-132.25
Total	-0.0470		-0.0478	100.00

^a^“Others” included unemployment and out of labour market

Our study has several limitations. First, we did not consider specific intervals for screening modality for colorectal cancer. Although the guideline recommends people to take FOBT every year, to have a sigmoidoscopy within five years, or a colonoscopy within 10 years [17], only KNHANES cycle 4 (2007) and cycle 5 (2010–2012) include information about types of colorectal cancer screening [21]. Second, we compared the lowest-income quintile with the highest-income quintile under the premise that the poor would experience an increase in the uptake of screening as much as the rich do. Although there might be a problem of over-screening among the rich, our premise would be still valid because this study used a survey question asking individual’s participation in screening services over the past two years as the outcome measure, not the number of participation [54].

## Conclusions

This study provides evidence on changes in socioeconomic inequalities in participation in gastric and colorectal cancer screening in Korea over the past decade, and shows decreases in inequalities for both cancer screenings. Despite policy reducing out-of-pocket payment for cancer screening possibly contributing to observed decreases in inequality, it alone is not likely to eliminate inequalities. Further research needs to identify barriers that prevent low-income people from the uptake of cancer screening despite free cancer screening program for all individuals who are eligible for Korea’s National Health Insurance.

## Additional file

Additional file 1: Table S1. Basic characteristics of respondents by participation of colorectal cancer screening^§^, KNHANES cycle 1 (1998), cycle 3 (2005) and cycle 5 (2010-2012). **Table S2.** Basic characteristics of the respondents by participation of gastric cancer screening^§^, KNHANES cycle 1 (1998), cycle 3 (2005) and cycle 5 (2010-2012). (DOCX 31 kb)
