# Supplementary material for: Assessment of trends in socioeconomic inequalities in cancer screening services in Korea, 1998–2012
Source: Int J Equity Health. 2016 Feb 24;15:30. doi: 10.1186/s12939-016-0319-7 (PMC4765127; doi:10.1186/s12939-016-0319-7)
Supplement: Additional file 1: Table S1. — Basic characteristics of respondents by participation of colorectal cancer screening§, KNHANES cycle 1 (1998), cycle 3 (2005) and cycle 5 (2010-2012). Table S2. Basic characteristics of the respondents by participation of gastric cancer screening§, KNHANES cycle 1 (1998), cycle 3 (2005) and cycle 5 (2010-2012). (DOCX 31 kb) [file 12939_2016_319_MOESM1_ESM.docx]

**Appendix**

Table 1. Basic characteristics of respondents by participation of colorectal cancer screening^§^, KHNANES cycle 1 (1998), cycle 3 (2005) and cycle 5 (2010-2012)

| Colorectal cancer screening | | Cycle 1 (1998) | | Cycle 3 (2005) | | Cycle 5 (2010-2012) | |
| --- | --- | --- | --- | --- | --- | --- | --- |
|  |  | Yes (n/mean) | No (n/mean) | Yes (n/mean) | No (n/mean) | Yes (n/mean) | No (n/mean) |
| Sex | Female | 111 | 1506 | 240 | 1234 | 1902 | 2898 |
|  | Male | 103 | 1157 | 235 | 901 | 1654 | 2006 |
| Age |  | 61.13 | 62.78 | 62.13 | 63.42 | 63.20 | 65.34 |
| Marital status | Single | 51 | 738 | 126 | 657 | 588 | 1263 |
|  | Married | 163 | 1925 | 349 | 1478 | 2968 | 3641 |
| Educational attainment | Elementary school | 112 | 1836 | 234 | 1263 | 1458 | 2667 |
|  | Middle school | 29 | 367 | 107 | 322 | 666 | 791 |
|  | High school | 53 | *347* | 90 | 397 | 945 | 989 |
|  | University or above | 20 | 113 | 44 | 153 | 487 | 457 |
| Employment status | Manual | 95 | 1399 | 234 | 906 | 1541 | 2125 |
|  | Non-manual | 14 | 75 | 34 | 101 | 325 | 307 |
|  | Others^µ^ | 105 | 1189 | 207 | 1128 | 1690 | 2472 |
| Income | Quintile 1 (lowest) | 37 | 712 | 76 | 524 | 519 | 1179 |
|  | Quintile 2 | 37 | 435 | 116 | 524 | 725 | 1076 |
|  | Quintile 3 | 52 | 752 | 65 | 292 | 733 | 850 |
|  | Quintile 4 | 25 | 281 | 104 | 392 | 792 | 959 |
|  | Quintile 5 (highest) | 63 | 483 | 114 | 403 | 787 | 840 |
| Health insurance | Medicaid | 10 | 147 | 18 | 148 | 84 | 210 |
|  | National Health Insurance | 204 | 2490 | 457 | 1979 | 3470 | 4692 |
|  | Neither^*^ | 0 | 26 | 0 | 8 | 2 | 2 |
| Region | Metro Seoul areas | 122 | 1803 | 302 | 1389 | 1957 | 2924 |
|  | Non-metro Seoul areas | 92 | 860 | 173 | 746 | 1599 | 1980 |
| Place of residence | Urban | 135 | 1272 | 315 | 1468 | 2698 | 858 |
|  | Rural | 79 | 1391 | 160 | 667 | 3373 | 1531 |
| Self-rated health | Good | 93 | 1059 | 214 | 902 | 834 | 1464 |
|  | Fair | 50 | 631 | 149 | 690 | 1677 | 2077 |
|  | Poor | 71 | 973 | 112 | 543 | 1045 | 1363 |

^§^ Respondents over the age of 50 were included for colorectal cancer screening service.

^*^This was excluded in decomposition analysis because none of them participated in colorectal cancer screening in cycle1 and cycle 3.

^µ^ “Others” included unemployment and out of labour market

Table 2. Basic characteristics of the respondents by participation of gastric cancer screening^§^, KHNANES cycle 1 (1998), cycle 3 (2005) and cycle 5 (2010-2012)

| Gastric cancer screening | | Cycle 1 (1998) | | Cycle 3 (2005) | | Cycle 5 (2010-2012) | |
| --- | --- | --- | --- | --- | --- | --- | --- |
|  |  | Yes (n/mean) | No (n/mean) | Yes (n/mean) | No (n/mean) | Yes (n/mean) | No (n/mean) |
| Sex | Female | 331 | 2285 | 808 | 1724 | 3725 | 3262 |
|  | Male | 329 | 1874 | 682 | 1366 | 2899 | 2388 |
| Age |  | 53.22 | 55.66 | 55.40 | 55.66 | 58.25 | 59.78 |
| Marital status | Single | 97 | 840 | 289 | 750 | 971 | 1294 |
|  | Married | 563 | 3319 | 1201 | 2340 | 5653 | 4356 |
| Educational attainment | Elementary school | 248 | 2147 | 508 | 1194 | 2165 | 2320 |
|  | Middle school | 121 | 774 | 271 | 501 | 1022 | 818 |
|  | High school | 182 | *934* | 427 | 968 | 2035 | 1534 |
|  | University or above | 109 | 304 | 284 | 427 | 1402 | 978 |
| Occupation | Manual | 321 | 2336 | 693 | 1533 | 2871 | 2441 |
|  | Non-manual | 101 | 269 | 232 | 298 | 1045 | 692 |
|  | Others^µ^ | 238 | 1554 | 565 | 1259 | 2708 | 2517 |
| Income | Quintile 1 (lowest) | 84 | 978 | 237 | 688 | 1128 | 1352 |
|  | Quintile 2 | 128 | 910 | 372 | 801 | 1373 | 1306 |
|  | Quintile 3 | 103 | 696 | 209 | 449 | 1218 | 988 |
|  | Quintile 4 | 232 | 1145 | 281 | 658 | 1495 | 1138 |
|  | Quintile 5 (highest) | 113 | 430 | 391 | 494 | 1410 | 866 |
| Insurance | Medicaid | 15 | 173 | 73 | 146 | 160 | 224 |
|  | National Health Insurance | 644 | 3939 | 1416 | 2931 | 6463 | 5422 |
|  | Neither | 1 | 47 | 1 | 13 | 1 | 4 |
| Region | Metro Seoul areas | 389 | 2721 | 867 | 1862 | 3611 | 3127 |
|  | Non-metro Seoul areas | 271 | 1438 | 623 | 1228 | 3013 | 2523 |
| Place of residence | Urban | 436 | 2306 | 1116 | 2320 | 5054 | 1570 |
|  | Rural | 224 | 1853 | 374 | 770 | 4178 | 1472 |
| Self-rated health | Good | 234 | 1344 | 462 | 951 | 1384 | 1468 |
|  | Fair | 208 | 1223 | 555 | 1122 | 3137 | 2529 |
|  | very poor | 218 | 1592 | 473 | 1017 | 2103 | 1653 |

^§^Respondents over the age of 40 were included for gastric cancer screening service.

^µ^ “Others” included unemployment and out of labour market
